# Hsa-miR-100-3p Controls the Proliferation, DNA Synthesis, and Apoptosis of Human Sertoli Cells by Binding to SGK3

**DOI:** 10.3389/fcell.2021.642916

**Published:** 2021-05-11

**Authors:** Bang Liu, Yinghong Cui, Wei Chen, Li Du, Chunyun Li, Cailin Wan, Zuping He

**Affiliations:** The Key Laboratory of Model Animals and Stem Cell Biology in Hunan Province, School of Medicine, Hunan Normal University, Changsha, China

**Keywords:** human Sertoli cells, hsa-miR-100-3p, proliferation, apoptosis, SGK3

## Abstract

Human Sertoli cell is required for completing normal spermatogenesis, and significantly, it has important applications in reproduction and regenerative medicine because of its great plasticity. Nevertheless, the molecular mechanisms underlying the fate decisions of human Sertoli cells remain to be clarified. Here, we have demonstrated the expression, function, and mechanism of Homo sapiens-microRNA (hsa-miR)-100-3p in human Sertoli cells. We revealed that miR-100-3p was expressed at a higher level in human Sertoli cells by 10% fetal bovine serum (FBS) than 0.5% FBS. MiR-100-3p mimics enhanced the DNA synthesis and the proliferation of human Sertoli cells, as indicated by 5-ethynyl-2′-deoxyuridine (EdU) and Cell Counting Kit-8 (CCK-8) assays. Flow cytometry showed that miR-100-3p mimics reduced the apoptosis of human Sertoli cells, and notably, we predicted and further identified serum/glucocorticoid regulated kinase family member 3 (SGK3) as a direct target of MiR-100-3p. SGK3 silencing increased the proliferation and decreased the apoptosis of human Sertoli cells, while SGK3 siRNA 3 assumed a similar role to miR-100-3p mimics in human Sertoli cells. Collectively, our study indicates that miR-100-3p regulates the fate decisions of human Sertoli cells by binding to SGK3. This study is of great significance, since it provides the novel epigenetic regulator for the proliferation and apoptosis of human Sertoli cells and it may offer a new clue for gene therapy of male infertility.

## Introduction

Abnormal spermatogenesis causes male infertility. Sertoli cell is one of the most important somatic cells within the testis because it is essential for regulating normal spermatogenesis and male germ cell development. In anatomical structure, the Sertoli cell is surrounded by male germ cells in the seminiferous tubules, and it directly contacts with male germ cells of different stages. Sertoli cell is tightly linked by multiprotein complexes that constitute the blood–testis barrier (BTB), which protects male germ cells from immunological rejection (Shi et al., 2018). Besides, Sertoli cell can provide a number of growth factors for the signaling transduction of male germ cells (Hofmann, 2008), e.g., bone morphogenetic protein 4 (BMP4), stem cell factor (SCF), glial cell-derived neurotrophic factor (GDNF), and fibroblast growth factor 2 (FGF2). Androgen receptor (AR) is specifically expressed in Sertoli cell, and its functional failure leads to immature Sertoli cells and spermatogenesis arrest (Singh et al., 1995; Meachem et al., 1997; Meroni et al., 2019). Finally, Sertoli cells can remove apoptotic germ cells with an aim to retain the homeostasis of the testis (Gong et al., 2018). Significantly, recent studies demonstrate that Sertoli cells have the potential to become the cells of other lineages, including the induced pluripotent stem (iPS) cells, neural stem cells (Alamoudi et al., 2018), and Leydig cells (Fu et al., 2019), reflecting that Sertoli cells might have significant applications in cell transplantation and tissue engineering for human diseases.

MicroRNA (known as the miRNA) has been demonstrated to play essential roles in mediating cellular proliferation, differentiation, transdifferentiation, and apoptosis. We have recently demonstrated that a number of miRNAs regulate the fate decisions of human spermatogonial stem cell (SSC). For example, miR-1908-3p controls human SSC renewal and apoptosis by binding to Kruppel-like factor 2 (KLF2) (Chen et al., 2020), while miR-122-5p regulates human SSC fate decisions *via* targeting casitas B-lineage lymphoma (CBL) (Zhou et al., 2020). Moreover, we have revealed that P21-activated kinase 1 (PAK1)/miR-31-5p controls human SSC proliferation and the apoptosis *via* targeting juxtaposed with another zinc finger protein 1 (JAZF1) (Fu et al., 2019), and miR-663a mediates human SSC proliferation and apoptosis by targeting transcription factor nuclear factor I X-type (NFIX) (Zhou et al., 2018). We have also compared the global miRNA profiles in human spermatogonia, pachytene spermatocytes, and spermatids between normal men and non-obstructive azoospermia patients (Yao et al., 2017). Nevertheless, the roles and molecular mechanisms of miRNAs in mediating human Sertoli cell remain elusive. It has been reported that miR-638 suppresses the growth of immature Sertoli cells by regulating sperm-associated antigen 1 (SPAG1) (Hu et al., 2017). MiR-130a has been shown to stimulate the proliferation of the immature porcine Sertoli cells *via* the activation of SMAD5 (Luo et al., 2020), while miR-320-3p is specifically expressed in mouse Sertoli cells and it reduces the lactate production of Sertoli cells through suppressing the level of glucose transporter 3 (Zhang et al., 2018). Recently, we have revealed the expression and the effect of miR-202-3p on regulating human Sertoli cells (Yang et al., 2019). These studies indicate that miRNA may be involved in controlling the fate determinations of Sertoli cells. It is well known that 10% fetal bovine serum (FBS) can significantly promote the proliferation of human Sertoli cells. Therefore, we compared the miRNA expression profiles in human Sertoli cells between 10% FBS and 0.5% FBS with an aim to identify novel miRNAs that could promote the proliferation of human Sertoli cells, and we revealed that miR-100-3p was expressed at a higher level in human Sertoli cells by 10% FBS than 0.5% FBS. In this study, we have uncovered that has-miR-100-3p promotes the proliferation and DNA synthesis and that it inhibits the apoptosis of human Sertoli cells by binding to serum/glucocorticoid regulated kinase family member 3 (SGK3). Thus, this study offers new epigenetic mechanisms controlling human Sertoli cell fate decisions, and importantly, it might provide new biomarkers for the treatment of male infertility.

## Materials and Methods

### Culture of Human Sertoli Cells

We isolated human Sertoli cells from six obstructive azoospermia (OA) patient testicular tissues by the two steps enzymatic digestion followed by differential plating (Yang et al., 2019). Human Sertoli cells were seeded onto the culture dish, and they were cultured with Dulbecco’s modified Eagle’s medium (DMEM)/F12 (Gibco, United States) by the addition of 10% FBS (Gibco, United States) and 1% penicillin and streptomycin (Gibco, United States) at 34°C in 5% CO_2_ incubator. This study was approved by the Institutional Ethical Review Committee of Hunan Normal University, and an informed consent of testis tissues for research only was obtained from each OA patient.

### RNA Extraction and RT-PCR

We extracted total RNA from human Sertoli cells when they were cultured with 10% or 0.5% FBS for 3 h, respectively, by the RNAiso Plus reagent (Takara, Japan). NanoDrop (Thermo Fisher Scientific, United States) was utilized to determine the concentrations of total RNA, and RNA with good quality was employed for RT-PCR. We performed RT of RNA to obtain the cDNA by the First Strand cDNA Synthesis Kit (Thermo Fisher Scientific, United States) and PCR reaction of the cDNA in terms of the protocol (Yang et al., 2014). We chose the following gene primers, including Wilms’ tumor gene 1 (WT1), GATA binding protein 4 (GATA4), GDNF, SCF, follicle-stimulating hormone receptor (FSHR), SRY-related high-mobility group-box gene 9 (SOX9), AR, and FGF2, and the sequences of those genes were shown in Supplementary Table 1.

The PCR reactions of cDNA were completed for 35 cycles pursuant to the conditions we previously described (Yang et al., 2014). The PCR products were separated by electrophoresis with 2% agarose gels stained with Safer ethidium bromide Alternatives-GelGreen (Biotium, United States). Images were exposed to the Gel Documentation and Image Analysis System ChampGel 5000 (SageCreation, China). No cDNA but with PCR of glyceraldehyde 3-phosphate dehydrogenase (*GAPDH*) primers was used as a negative control.

### Real-Time qPCR

The RNAiso Plus reagent (Takara, Japan) was employed to isolate total RNA from human Sertoli cells and these cells without or with the treatment of miR-100-3p mimics, miR-100-3p inhibitor, miRNA mimics control, miRNA inhibitor control, SGK3 siRNAs, or siRNA control. RT reactions were performed for 60 min at 37°C using the mixture consisting of 100 ng RNA, 10 μl 2× TS miRNA Reaction Mix, 1 μl miRNA RT Enzyme Mix, and RNase-free water in a total volume of 20 μl, and heat inactivation of RT was done for 5 s at 85°C in a Veriti 96-Well Thermal Cycler (Bio-Rad, United States). Nuclease-free water was employed to dilute the RT reaction mixture by five times. The miRNA primer sequences for real-time qPCR were shown in Supplementary Table 1. Real-time qPCR was performed three times utilizing Power SYBR Green PCR Master Mix (Biosystems, United Kingdom) and a CFX Connect Real-Time System (Bio-Rad, United States), pursuant to the protocol described previously (Wang et al., 2017). The levels of miRNAs were normalized to U6, and their relative expression was calculated using the 2^–ΔΔ*Ct*^ method (Wang et al., 2017).

The levels of *SGK3* gene were measured by real-time qPCR in human Sertoli cells with treatment of miR-100-3p mimics, miRNA mimics control, miR-100-3p inhibitors, miRNA inhibitor control, SGK3-siRNAs, or siRNA control, pursuant to the protocol described previously (Wang et al., 2017). *SGK3* primers were designed and shown in Supplementary Table 1, and the relative levels of *SGK3* gene were calculated by the 2^–ΔΔ*Ct*^ method after normalization to housekeeping gene *GAPDH* (Wang et al., 2017).

### Immunocytochemistry

For the immunocytochemical staining, 4% paraformaldehyde (PFA; Beyotime, China) was used for fixation of human Sertoli cells for 20 min, and 0.5% Triton X-100 (Sigma, United States) was employed to permeabilize these cells for 15 min. These cells were blocked with 5% bovine serum albumin (BSA) for 90 min, and they were incubated with primary antibodies as shown in Supplementary Table 2 overnight at 4°C. Primary antibodies were replaced with isotype IgGs to serve as negative controls. The secondary antibody IgG (Sigma, United States) with 1:2,000 dilution was used to recognize the primary antibodies. Cell nuclei were labeled by 4′,6-diamidino-2-phenylindole (DAPI), while the images were screened by a fluorescence microscope (Leica, Germany).

### Transfection of MiR-100-3p Mimics, MiR-100-3p Inhibitors, or SGK3 siRNAs to Human Sertoli Cells

The mimics, inhibitors, and the controls for miR-100-3p were bought from GenePharma (Shanghai, China). The oligonucleotide sequences for miR-100-3p mimics and inhibitors were shown in Supplementary Table 3. Human Sertoli cells were planted to the culture dish at 1 × 10^5^ Sertoli cells/cm^2^ density, and they were cultured with DMEM/F12 containing the 10% FBS overnight. Human Sertoli cells were classified into four groups, namely, miR-100-3p mimics, miRNA mimics control, miR-100-3p inhibitor, and miRNA inhibitor control. Similarly, human Sertoli cells were categorized into four groups, including SGK3 siRNA 1, SGK3 siRNA 2, SGK3 siRNA 3, and the control siRNA. Transfection of miR-100-3p mimics, inhibitor, or SGK3 siRNA1-3 was completed, respectively, utilizing Lipofectamine 3000 transfection agent (Life Technologies, Carlsbad, CA, United States). Forty-eight or seventy-two hours after transfection, the cells were used for determining the mRNA or protein levels.

### Cell Counting Kit-8 Assay

For the proliferation assay, human Sertoli cells were plated onto 96-well microtiter plates (Corning, United States) at a density of 2,000 Sertoli cells/well, and they were transfected with miR-100-3p mimics, miRNA mimics control, miR-100-3p inhibitor, miRNA inhibitor control, SGK3 siRNA 3, or the control siRNA. After 5 days of culture, Cell Counting Kit-8 (CCK-8) assay (Dojin Laboratories, Japan) was performed to measure human Sertoli cell proliferation pursuant to the manufacturer’s direction. The culture medium was changed with 10% CCK-8 solution (Dojin Laboratories, Kumamoto, Japan), and the cells were incubated with it for 3 h. The absorbance was determined at 450 nm by a microplate reader.

### Western Blots

Human Sertoli cells were transfected with miR-100-3p mimics, miRNA mimics control, miR-100-3p inhibitor, miRNA inhibitor control, SGK3 siRNAs, or the control siRNA, and the RIPA buffer (Beyotime Biotechnology, China) was used for protein extraction from these cells. Centrifugation of cell lysates was performed at 12,000 × g for 20 min, while the protein concentrations were determined by bicinchoninic acid (BCA) kit (Dingguo, China). Thirty micrograms of cell lysate were employed for Western blots using the antibodies (Supplementary Table 2) in terms of the method (He et al., 2007), while the blots were detected with chemiluminescence (SageCreation, China).

### 5-Ethynyl-20-Deoxyuridine (EdU) Incorporation Assay

In total, 3,000 human Sertoli cells/well were plated onto 96-well plates (Corning, United States) in DMEM/F12 with the addition of 50 mM 5-ethynyl-2′-deoxyuridine (EdU; RiboBio, China). These cells were treated with miRNA mimics control, miR-100-3p mimics, miRNA inhibitor control, miR-100-3p inhibitor, SGK3 siRNA 3, or the control siRNA. We fixed the cells after 12 h of culture with 4% PFA, and they were neutralized by 1.8 mg/ml glycine and permeabilized with 0.5% Triton X-100 for 15 min. Apollo staining reaction buffer was utilized for EdU immunostaining, while Hoechst 33342 was employed for labeling cell nuclei. The EdU-positive cells were calculated from at least 500 cells by fluorescence microscopy (Leica, Germany).

### Annexin V and Propidium Iodide Staining and Flow Cytometry

The apoptosis of human Sertoli cells was examined by the allophycocyanin (APC) Annexin V and propidium iodide (PI) apoptosis detection kit (BioLegend, London, United Kingdom) and flow cytometry (BD, United States) after transfecting without or with the treatment of miR-100-3p mimics, miR-100-3p inhibitors, miRNA mimics control, miRNA inhibitor control, SGK3 siRNAs, or the control siRNA. Human Sertoli cells were seeded onto six-well plates (Corning, United States) at a density of 5 × 10^4^ cells/well, and they were collected by centrifuging at 1,000 rpm for 5 min. Meanwhile, the cells were labeled by the non-vital dye PI and Annexin V-fluorescein isothiocyanate (FITC), which was employed to detect different cell populations, including the intact cells (i.e., FITC^–^PI^–^ cells), the early apoptotic cells (i.e., FITC^+^PI^–^ cells), and the late apoptotic cells (i.e., FITC^+^PI^+^ cells).

### Terminal Deoxynucleotidyl Transferase dUTP Nick End Labeling Assay

The terminal deoxynucleotidyl transferase dUTP nick end labeling (TUNEL) Apoptosis Detection Kit (Yeasen, China) was utilized to further examine the apoptotic cells. In total, 5 × 10^4^ human Sertoli cells/well were treated with miRNA mimics control, miR-100-3p mimics, miRNA inhibitor control, miR-100-3p inhibitor, SGK3 siRNA 3, or the control siRNA, and 4% PFA was used for fixing these cells for 30 min at 4°C. After extensive washes by phosphate buffered saline (PBS), the cells were treated with 20 mg/ml Proteinase K, 1× DNaseI Buffer for 6 min and 10 U/ml DNaseI for 10 min, and they were washed with deionized water and incubated with 1× Equilibration Buffer for 30 min. These cells were stained with Alexa Fluor 647 in buffer mixed with TdT Enzyme for 70 min at 37°C. After extensive washes by PBS, DAPI was used for labeling the cell nuclei, and the TUNEL-positive cells were counted by the fluorescence microscope (Leica, Germany).

### Dual Luciferase Assay

A total of 3,000 human Sertoli cells were seeded onto 96-well plates (Corning, United States). After 24 h of culture, Lipofectamine 3000 transfection agent (Sigma, United States) was employed to transfect miR-100-3p mimics or the mimics control to these cells. Forty-eight hours later, human Sertoli cells were transfected with 500 ng plasmids containing the binding sequence in 3′ untranslated regions (UTRs) of SGK3, firefly luciferase (reporter), or the renilla luciferase (internal control) (Genecreate, China) using the Lipofectamine 3000 reagent (Sigma, United States). Forty-eight hours after transfection, human Sertoli cells were lysed, and luciferase activity was determined using the 96-well plate luminometer (Corning, United States). The results were normalized to cells transfected with miRNA mimics control.

### Statistical Analysis

All results were shown as the mean ± SEM. Comparisons between two groups were performed using the unpaired *t*-test, and *P*-value < 0.05 was regarded as statistically significant. Each experiment was conducted at least three times independently.

## Results

### Biochemical Phenotype of Primary Human Sertoli Cells

**FIGURE 1:**
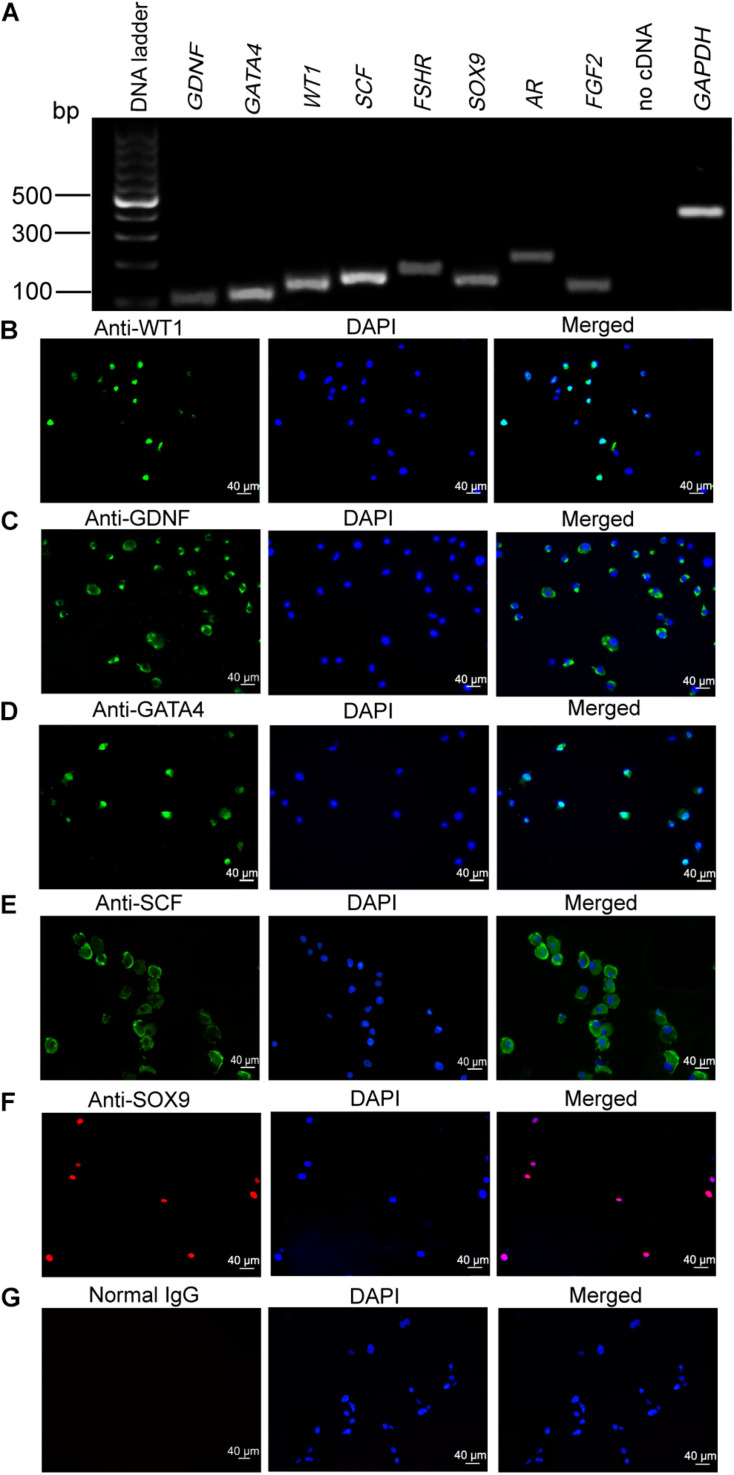
Phenotype of human Sertoli cells. **(A)** The transcripts of glial cell-derived neurotrophic factor (*GDNF*), GATA binding protein 4 (*GATA4*), Wilms’ tumor gene 1 (*WT1*), stem cell factor (*SCF*), follicle-stimulating hormone receptor (*FSHR*), SRY-related high-mobility group-box gene 9 (*SOX9*), androgen receptor (*AR*), and fibroblast growth factor 2 (*FGF2*) were detected by RT-PCR in human Sertoli cells. Housekeeping gene glyceraldehyde 3-phosphate dehydrogenase (*GAPDH*) was used to be the RNA loading control. **(B–G)** Immunocytochemistry revealed the positive cells of WT1 **(B)**, GDNF **(C)**, GATA4 **(D)**, SCF **(E)**, SOX9 **(F)**, and isotype IgGs **(G)** in human Sertoli cells. Scale bar in panels **(B–G)** = 40 μm.

We first verified the identity of the cells used in this study using numerous markers for human primary Sertoli cell. The transcripts of *GDNF*, *GATA4*, *WT1*, *SCF*, *FSHR*, *SOX9*, *AR*, and *FGF2* were detected in the isolated human cells (Figure 1A) as shown by RT-PCR. No PCR product was detected in the no cDNA samples but with PCR by the primers of *GAPDH*. Furthermore, immunocytochemistry showed that the cells were positively stained for WT1 (Figure 1B), GDNF (Figure 1C), GATA4 (Figure 1D), SCF (Figure 1E), and SOX9 (Figure 1F). No immunostaining was seen in the cells when the isotype IgGs were used for the replacement of primary antibodies (Figure 1G), which verified the specific expression of these proteins mentioned above.

### Differential Expression of MiR-100-3p in Human Sertoli Cells Between 10% and 0.5% Fetal Bovine Serum

**FIGURE 2:**
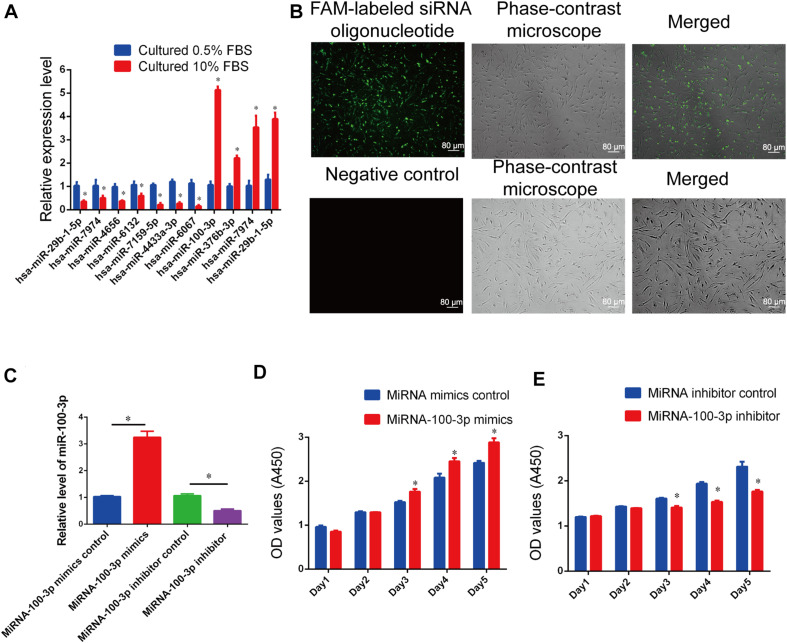
MiR-100-3p controls human Sertoli cell proliferation. **(A)** Real-time qPCR showed the relative expression levels of Homo sapiens-microRNA (hsa-miR)-29b-1-5p, hsa-miR-7974, hsa-miR-4656, hsa-miR-6132, hsa-miR-7159-5p, hsa-miR-4433a-3p, hsa-miR-6067, hsa-miR-100-3p, hsa-miR-376b-3p, hsa-miR-7974, and hsa-miR-29b-1-5p in human Sertoli cells by 10% fetal bovine serum (FBS) compared with 0.5% FBS. **(B)** The transfection efficiency of miR-100-3p mimics and inhibitor was evaluated by fluorescence microscope and phase-contrast microscope. Scale bar = 80 μm. **(C)** The relative expression levels of miR-100-3p in human Sertoli cells treated with miR-100-3p mimics or inhibitor. **(D,E)** The cell growth of human Sertoli cells treated with miR-100-3p mimics **(D)** and miR-100-3p inhibitor **(E)** for 5 days. * denoted the statistical difference between miR-100-3p mimics and miRNA mimics control or miR-100-3p inhibitor and miRNA inhibitor control.

We have found that FBS promotes human Sertoli cell proliferation. To seek novel miRNAs that are involved in the DNA synthesis and proliferation of human Sertoli cells, we compared the differences in the global miRNA profiles of human Sertoli cells cultured in different serum concentrations (10% vs. 0.5% FBS) using miRNA microarrays. The representative miRNAs that were upregulated and downregulated in human Sertoli cells cultured with 10% FBS compared to 0.5% FBS were identified and listed in Supplementary Table 4. Among the miRNAs with differential expression (fold changes ≥ 1.5 and *p* < 0.05), miR-100-3p was expressed at a higher level in human Sertoli cells by 10% FBS than 0.5% FBS. Real-time qPCR reflected the highest level of miR-100-3p in human Sertoli cells by 10% FBS compared with 0.5% FBS (Figure 2A), which was consistent with miR-10-3p expression in human Sertoli cells affected by 10% FBS and 0.5% FBS, as detected by miRNA microarrays. These data suggest that miR-100-3p is involved in regulating the proliferation of human Sertoli cells.

### MiR-100-3p Stimulates Human Sertoli Cell Growth and DNA Synthesis

We examined the role of miR-100-3p in regulating human Sertoli cells using miR-100-3p mimics and inhibitors. As shown in Figure 2B, the transfection efficiency of miR-100-3p mimics or inhibitors was more than 80% in human Sertoli cells. MiRNA mimics control, miR-100-3p mimics, miRNA inhibitor control, or miR-100-3p inhibitor was transfected to human Sertoli cells by Lipofectamine 3000. Twenty-four hours after transfection, real-time qPCR demonstrated that the level of miR-100-3p was upregulated by miR-100-3p mimics in human Sertoli cells when compared to miRNA mimics control (Figure 2C). In contrast, miR-100-3p inhibitor reduced miR-100-3p expression in human Sertoli cells compared to miRNA inhibitor control (Figure 2C).

**FIGURE 3:**
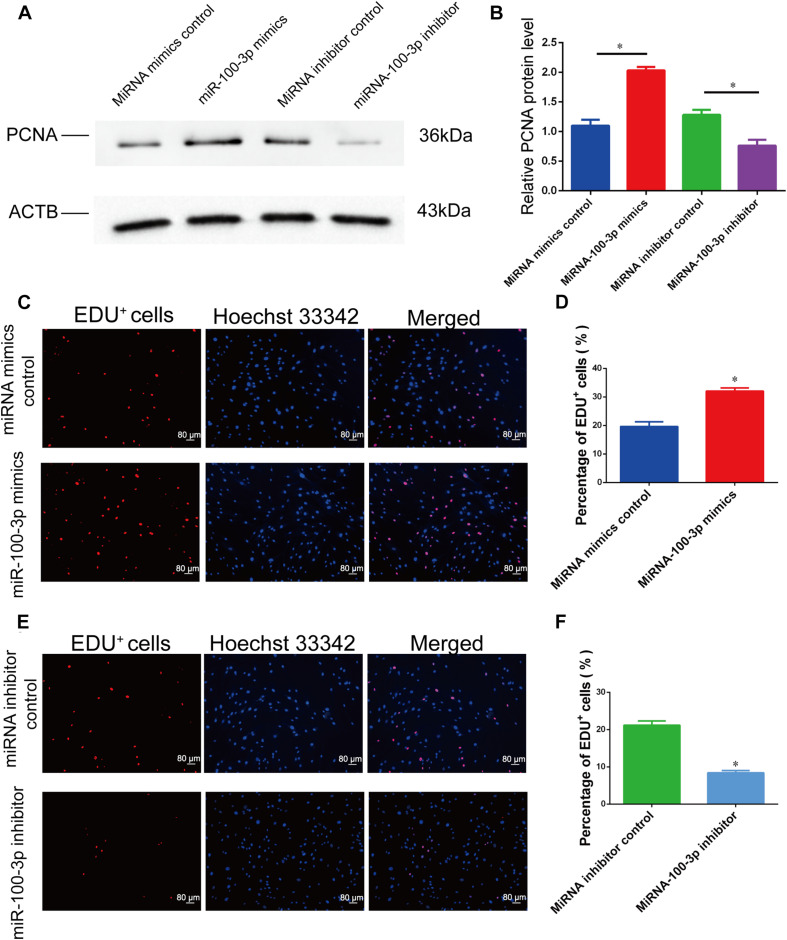
MiR-100-3p affects human Sertoli cell DNA synthesis. **(A)** Proliferating cell nuclear antigen (PCNA) expression was shown by Western blots in human Sertoli cells at 72 h after miR-100-3p mimics or inhibitor transfection. **(B)** The relative expression of PCNA in human Sertoli cells at 72 h after miR-100-3p mimics and inhibitor transfection. **(C–F)** The ratios of 5-ethynyl-2′-deoxyuridine (EdU)-positive cells were illustrated by EdU incorporation assays in human Sertoli cell treatment with miRNA mimics control vs. the miR-100-3p mimics **(C,D)** as well as miR-100-3p inhibitor vs. miRNA inhibitor **(E,F)**. Scale bar in panels **(C,E)** = 80 μm. * indicated the statistical difference between miR-100-3p mimics and miRNA mimics control or miR-100-3p inhibitor and miRNA inhibitor control.

The influence of miR-100-3p on human Sertoli cell proliferation was measured by various kinds of methods. Cell proliferation assays were performed from 1 day to 5 days after transfection of miR-100-3p mimics and miR-100-3p inhibitors, and miR-100-3p mimics increased the numbers of human Sertoli cells compared to miRNA mimics control (Figure 2D), whereas miR-100-3p inhibitor reduced the growth of human Sertoli cells when compared with miRNA inhibitor control (Figure 2E). The levels of proliferating cell nuclear antigen (PCNA) protein were increased by miR-100-3p mimics and decreased by miR-100-3p inhibitor, as shown by Western blots (Figures 3A,B). The percentages of EdU-positive cells were enhanced by miR-100-3p mimics (Figures 3C,D) and decreased by miR-100-3p inhibitor (Figures 3E,F), as indicated by EdU incorporation assay. Collectively, these results implicate that miR-100-3p can stimulate human Sertoli cell growth and DNA synthesis.

### MiR-100-3p Inhibits Human Sertoli Cell Apoptosis

**FIGURE 4:**
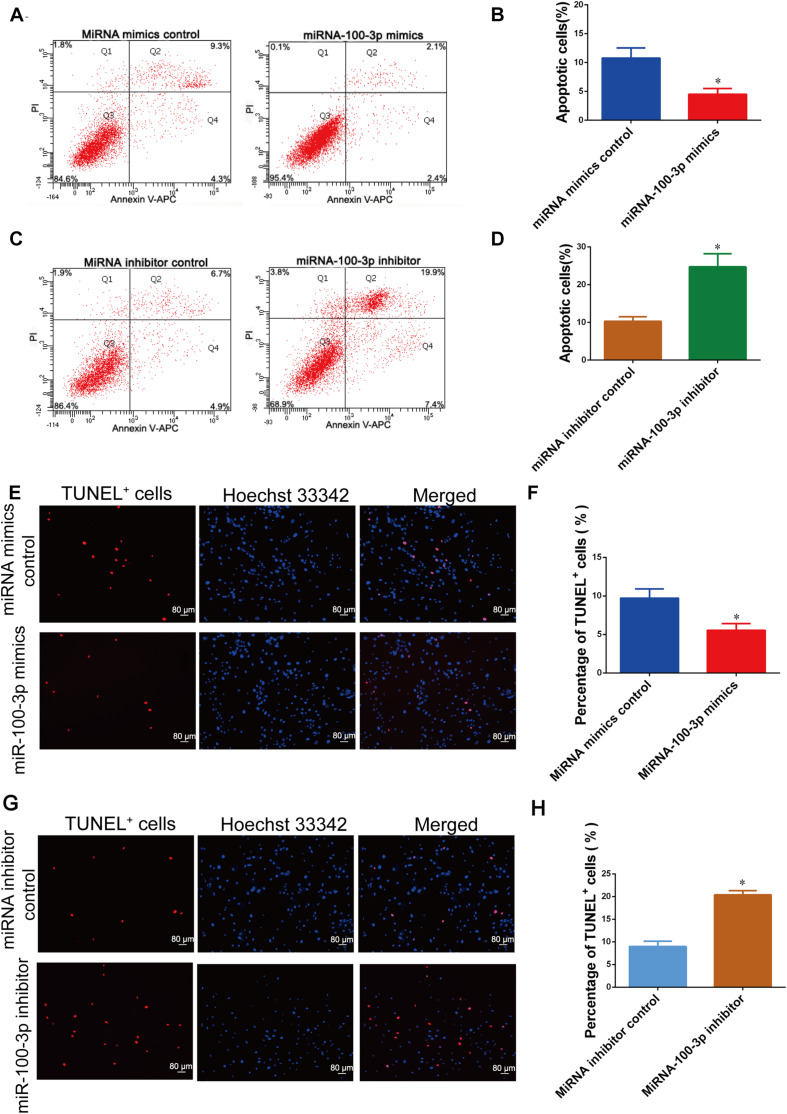
MiR-100-3p inhibits human Sertoli cell apoptosis. **(A–D)** The percentages of human Sertoli cell early and late apoptosis affected by miR-100-3p mimics **(A,B)** and miR-100-3p inhibitor **(C,D)** by comparison to the respective control. **(E–H)** The ratios of terminal deoxynucleotidyl transferase dUTP nick end labeling (TUNEL)-positive cells in human Sertoli cells treated with miR-100-3p mimics and miR-100-3p inhibitor by comparison to the respective control. Scale bar in panels **(E,G)** = 80 μm. * denoted the statistical difference between miR-100-3p mimics and miRNA mimics control or miR-100-3p inhibitor and miRNA inhibitor control.

We further determined the influence of miR-100-3p on human Sertoli cell apoptosis. As shown by APC Annexin V and PI staining and analyzed by flow cytometry, miR-100-3p mimics decreased human Sertoli cell apoptosis vs. miRNA mimics control (Figures 4A,B). By contrast, the percentage of human Sertoli cell apoptosis was enhanced by the miR-100-3p inhibitor compared to miRNA inhibitor control (Figures 4C,D). The percentages of TUNEL-positive cells were decreased by miR-100-3p mimics and enhanced by miR-100-3p inhibitor, as shown by TUNEL assay (Figures 4E,F,G,H). Therefore, our results indicate that miR-100-3p inhibits human Sertoli cell apoptosis.

### SGK3 Is a Direct Target of MiR-100-3p in Human Sertoli Cells

**FIGURE 5:**
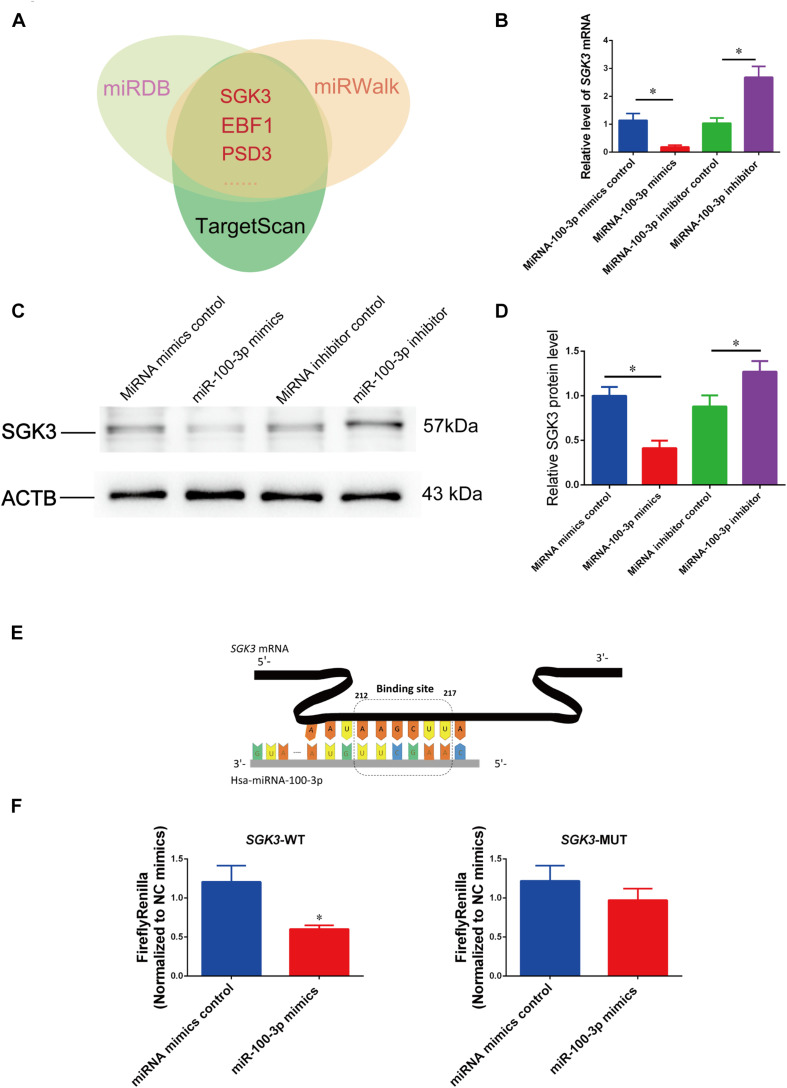
MiR-100-3p binds to serum/glucocorticoid regulated kinase family member 3 (SGK3) in human Sertoli cells. **(A)** MiR-100-3p targets were predicted by three bioinformatics tools. **(B)** The relative levels of *SGK3* mRNA in human Sertoli cells affected by miR-100-3p mimics or miR-100-3p inhibitor by comparison to the respective control. **(C,D**) The relative level of SGK3 in human Sertoli cells at day 3 after transfection of miR-100-3p mimics or miR-100-3p inhibitor after normalization to ACTB (beta-actin). **(E)** Schematic diagram illustrated the binding site of miR-100-3p to *SGK3* mRNA. **(F)** The binding of miR-100-3p to wild-type SGK3 by dual luciferase assays in human Sertoli cells after transfection of miR-100-3p mimics or the miRNA mimics control. * indicated the statistical difference between miR-100-3p mimics and miRNA mimics control or miR-100-3p inhibitor and miRNA inhibitor control.

We next sought to find miR-100-3p targets in controlling human Sertoli cells. Because miRNAs act *via* their seed sequence through the base-pair binding to the 3′ UTR of mRNAs. We used various kinds of miRNA prediction software (TargetScan, miRwalk, and miRDB) and predicted that SGK3, EBF1, and PSD3 were the potential targets for miR-100-3p (Figure 5A).

To determine whether SGK3 is a binding target for miR-100-3p in human Sertoli cells, we performed real-time qPCR and Western blots showing that *SGK3* transcripts were reduced by miR-100-3p mimics but increased by miR-100-3p inhibitors (Figure 5B), while SGK3 protein was diminished by miR-100-3p mimics but enhanced by miR-100-3p inhibitor (Figures 5C,D). The second to eighth nucleotides (known as the seed region) of miR-100-3p were able to bind to the 3′ UTR sequence of *SGK3* mRNA (Figure 5E), and the binding site of *SGK3* mRNA was further illustrated by the dual luciferase assay. The luciferase activity of the fusion genes was reduced by the sequence in 3′ UTR of *SGK3* mRNA in human Sertoli cells by miR-100-3p mimics (Figure 5F, left panel), whereas the mutated target sequences had no effect on the luciferase activity (Figure 5F, right panel). Considered together, these data indicate that SGK3 may be a direct target for miR-100-3p in human Sertoli cells.

### The Effect of Serum/Glucocorticoid Regulated Kinase Family Member 3 Knockdown on the Proliferation and Apoptosis of Human Sertoli Cells

**FIGURE 6:**
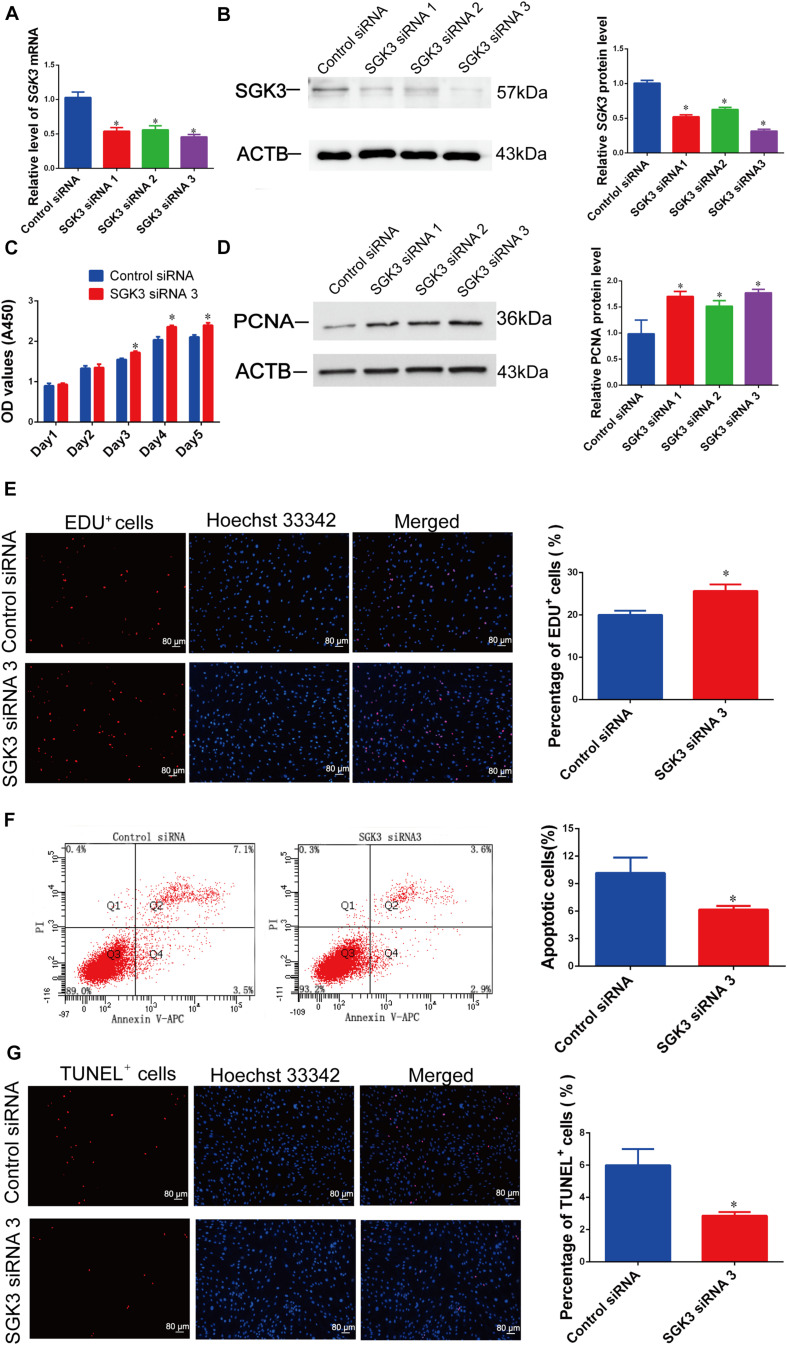
Serum/glucocorticoid regulated kinase family member 3 (SGK3) knockdown regulates human Sertoli cell proliferation, DNA synthesis, and apoptosis. **(A)** The relative levels of *SGK3* mRNA in human Sertoli cells after transfection of SGK3 siRNA 1–3 or the control siRNA. **(B)** The relative level of SGK3 protein in human Sertoli cells after transfection of SGK3 siRNA 1-3 or the control siRNA. **(C)** The cell growth of human Sertoli cells treated with SGK3 siRNA 3 and the control siRNA for 5 days. **(D)** The relative level of proliferating cell nuclear antigen (PCNA) protein in human Sertoli cells at day 3 transfected by SGK3 siRNA 1-3 or the control siRNA. **(E)** The 5-ethynyl-2′-deoxyuridine (EdU)-positive cells of human Sertoli cells treated with SGK3 siRNA 3 or the control siRNA. Scale bar in panel **(E)** = 80 μm. **(F)** The percentages of apoptosis in human Sertoli cells transfected with control siRNA or SGK3 siRNA 3. **(G)** The percentages of terminal deoxynucleotidyl transferase dUTP nick end labeling (TUNEL)-positive cells in human Sertoli cells transfected with control siRNA or SGK3 siRNA 3. Scale bar in panels **(E,G)** = 80 μm. * indicated the statistical difference between SGK3 siRNAs and the control siRNA.

We asked the impact of SGK3 on human Sertoli cell proliferation and apoptosis. We used SGK3 small interfering RNAs (siRNAs), including SGK3 siRNA 1–3, with different base-pair binding sites, to knock down the SGK3 level of human Sertoli cells. The transfection efficiency of SGK3 siRNAs in human Sertoli cells was more than 80%, as indicated by the transfection of FAM-labeled fluorescent oligo (Figure 2A). SGK3 siRNAs could knock down the levels of *SGK3* transcripts and proteins, while SGK3 siRNA 3 had the highest effectiveness for SGK3 silencing (Figures 6A,B).

SGK3 siRNA 3 caused the enhancement of human Sertoli cell number from 24 to 120 h of culture (Figure 6C). The PCNA protein level (Figure 6D) and the ratio of EdU-positive cells (Figure 6E) of human Sertoli cells were increased by SGK3 siRNAs, especially by SGK3 siRNA 3. By contrast, SGK3 knockdown reduced the apoptosis of the human Sertoli cells (Figures 6F,G). Together, these results reflect that SGK3 silencing stimulates the proliferation and suppresses the apoptosis of human Sertoli cells, which was in accordance with the influence of miR-100-3p mimics.

### The Synergetic Effect of MiR-100-3p and Serum/Glucocorticoid Regulated Kinase Family Member 3 SiRNA 3 on the Apoptosis of Human Sertoli Cells

**FIGURE 7:**
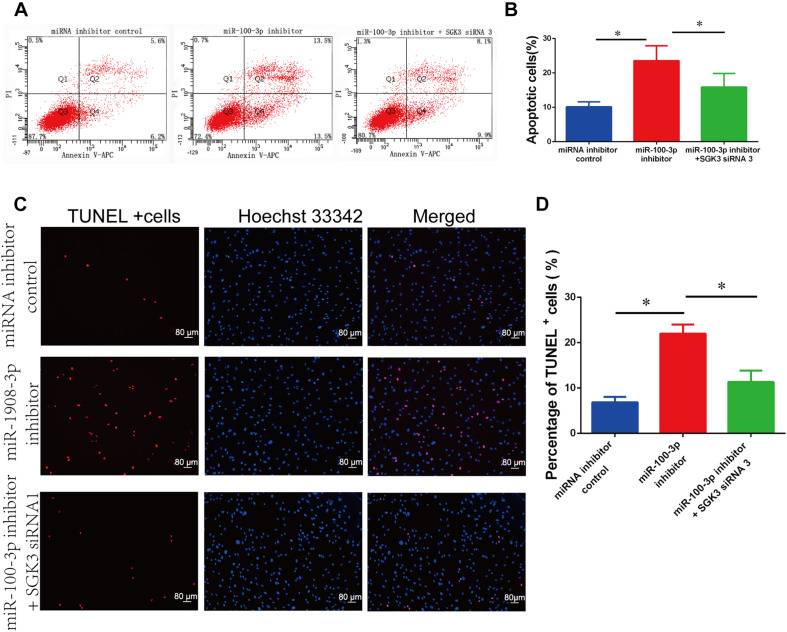
The impact of miR-100-3p and serum/glucocorticoid regulated kinase family member 3 (SGK3) siRNA 3 on the apoptosis of human Sertoli cells. **(A,B)** Allophycocyanin (APC) Annexin V/propidium iodide (PI) staining and flow cytometry showed the apoptotic percentages of human Sertoli cells affected by miRNA inhibitor control, miR-100-3p inhibitor, as well as miR-100-3p inhibitor and SGK3 siRNA 3. **(C,D)** Terminal deoxynucleotidyl transferase dUTP nick end labeling (TUNEL) assay revealed the percentages of TUNEL-positive cells in human Sertoli cells affected by miRNA inhibitor control, miR-100-3p inhibitor, as well as miR-100-3p inhibitor and SGK3 siRNA 3. Scale bar in panel **(C)** = 80 μm. * indicates the statistical differences between miR-100-3p inhibitor and SGK3 siRNA 3 and miR-100-3p inhibitor or between miR-100-3p inhibitor and miRNA inhibitor control.

We further inquired whether there was the synergetic effect of miR-100-3p inhibitor and SGK3 siRNA 3 on the apoptosis of human Sertoli cells. MiR-100-3p inhibitor and SGK3 siRNA 3 were co-transfected to these cells with Lipofectamine 3000 reagent. After 48 h of transfection, the number of early and late apoptosis was decreased in human Sertoli cells with the co-transfection of miR-100-3p inhibitor and SGK3 siRNA 3 compared with the cells transfected with miR-100-3p inhibitor (Figures 7A,B). TUNEL assay showed that the percentages of TUNEL-positive cells were decreased in human Sertoli cells co-transfected with miR-100-3p inhibitor and SGK3 siRNA 3 when compared to those cells transfected with miR-100-3p inhibitor (Figures 7C,D). These data implicate that there is a synergetic effect of miR-100-3p inhibitor and SGK3 siRNA 3 on the apoptosis of human Sertoli cells.

### The Synergetic Effect of MiR-100-3p and Serum/Glucocorticoid Regulated Kinase Family Member 3 SiRNA 3 on DNA Synthesis and Proliferation of Human Sertoli Cells

**FIGURE 8:**
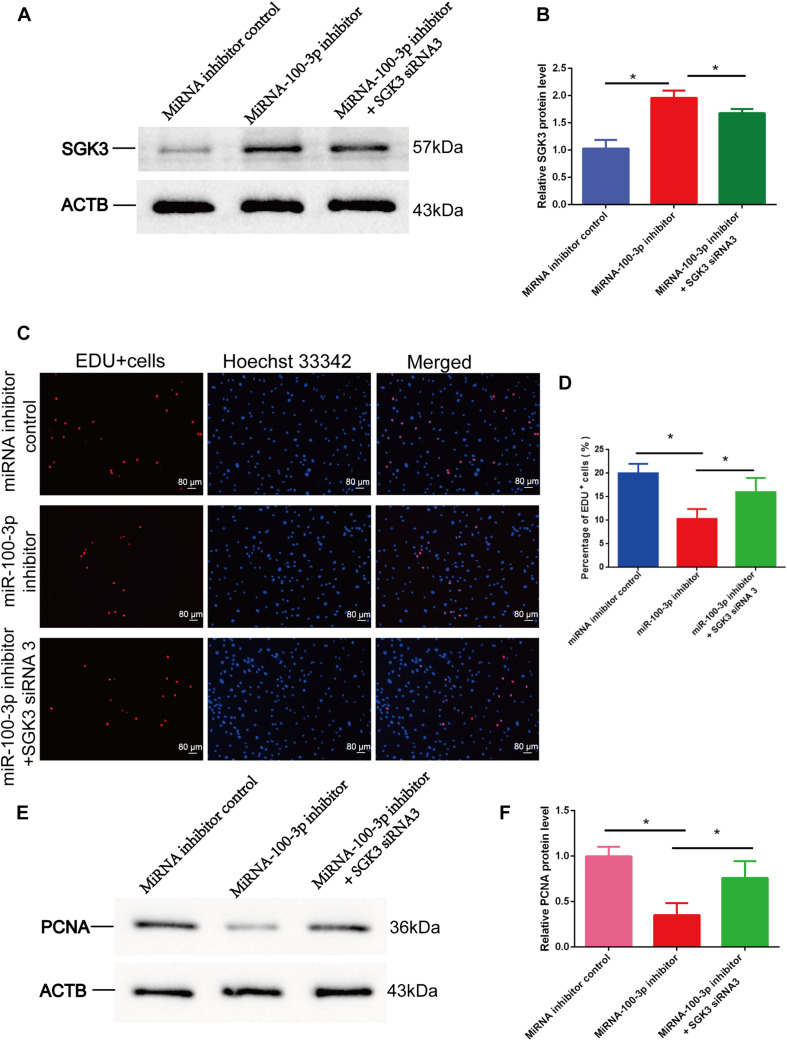
The effect of miR-100-3p and serum/glucocorticoid regulated kinase family member 3 (SGK3) siRNA 3 on the DNA synthesis and proliferation of human Sertoli cells. **(A,B,E,F)** Western blots displayed the expression levels of SGK3 **(A,B)** and proliferating cell nuclear antigen (PCNA) **(E,F)** proteins in human Sertoli cells affected by miRNA inhibitor control, miR-100-3p inhibitor, as well as miR-100-3p inhibitor and SGK3 siRNA 3. **(C,D)** The 5-ethynyl-2′-deoxyuridine (EdU) incorporation assay revealed the percentages of EdU-positive cells in human Sertoli cells affected by miRNA inhibitor control, miR-100-3p inhibitor, as well as miR-100-3p inhibitor and SGK3 siRNA 3. Scale bar in panel **(C)** = 80 μm. * denoted the statistical differences between miR-100-3p inhibitor and SGK3 siRNA 3 and miR-100-3p inhibitor or between miR-100-3p inhibitor and miRNA inhibitor control.

We finally explored the influence of miR-100-3p inhibitor and SGK3 siRNA 3 on the DNA synthesis and proliferation of human Sertoli cells. Western blots showed that the expression levels of SGK3 (Figures 8A,B) and PCNA (Figures 8E,F) proteins were enhanced in human Sertoli cells with the co-transfection of miR-100-3p inhibitor and SGK3 siRNA 3 compared with the cells transfected with miR-100-3p inhibitor. Similarly, the EdU incorporation assay displayed that the percentages of EdU-positive cells were increased in human Sertoli cells co-transfected with miR-100-3p inhibitor and SGK3 siRNA 3 in comparison to these cells transfected with miR-100-3p inhibitor (Figures 8C,D). Together, these results indicate that there is the synergetic influence of miR-100-3p inhibitor and SGK3 siRNA 3 on the DNA synthesis and proliferation of human Sertoli cells.

## Discussion

Sertoli cell is required for regulating spermatogenesis because it provides the niche and the nutrition for the proliferation and differentiation of male germ cells (Franca et al., 2016). It has been shown that the amount of male germ cells is positively correlated with the number of Sertoli cells (Rebourcet et al., 2017). In addition, Sertoli cell can be reprogrammed to become Leydig cell by Wt1 ablation (Zhang et al., 2015) and neural stem cells (Alamoudi et al., 2018), highlighting that Sertoli cell has significant applications in male reproduction and cell therapy of various diseases. Although great progress has been made in understanding the biology of rodent Sertoli cells, the molecular mechanisms for human Sertoli cell fate decisions, especially epigenetic regulators, are still unknown.

By comparing the expression levels of miRNAs between 0.5% FBS and 10% FBS in human Sertoli cells, we revealed that miR-100-3p was enhanced by 10% FBS, as demonstrated by miRNA microarray and verified by real-time qPCR. MiR-100-3p has been shown to control the proliferation and the apoptosis of human gastric cancer cells *via* binding to bone morphogenic protein type 2 receptor (BMPR2) (Peng et al., 2019), and it may be involved in producing interleukin (IL)-8 and IL-1β in mesangial cells (Liang et al., 2016). In this study, we have shown that miR-100-3p stimulates human Sertoli cell proliferation and DNA synthesis, as determined by CCK-8 assay, PCNA expression, and EdU incorporation assay. We have uncovered that miR-100-3p suppresses human Sertoli cell apoptosis, as indicated by Annexin V and PI staining and flow cytometry as well as TUNEL assay.

Predicted by miRWalk and miRDB software, we assumed that SGK3 is a potential target. Furthermore, we uncovered that the level of SGK3 was reduced by miR-100-3p mimics and increased by miR-100-3p inhibitor, reflecting that SGK3 is the binding target for miR-100-3p in human Sertoli cells. Dual luciferase reporter assays further demonstrated that miR-100-3p is able to bind to SGK3 in human Sertoli cells. SGK3 knockdown led to changes in human Sertoli cell proliferation and apoptosis, which was consistent with the influence of the miR-100-3p mimics. SGK3, belonging to the SGK family of acylglycerol kinase (AGK) kinases, is expressed in many kinds of cells, especially in testis and pancreas, and it exerts broad functions (Lang et al., 2006). It has been shown that SGK3 plays a role as a carcinogen in breast cancer, ovarian cancer, and hepatocellular carcinoma, and it participates in controlling cell survival, differentiation, and material transport (Lang et al., 2006; Wang et al., 2019). SGK3 is composed of a 3′ UTR that is the target seed region of numerous miRNAs. The transcription and location of SGK3 are affected by many factors, e.g., miRNA-335-5p (Yao et al., 2018), and it is involved in many intracellular signaling transduction pathways, including phosphoinositide 3-kinase (PI3K)/AKT pathway (Basnet et al., 2018). In the current study, we identified SGK3 as a direct and binding target of miR-100-3p in human Sertoli cell fate decisions.

In conclusion, we have reported for the first time that miR-100-3p promotes DNA synthesis and proliferation and suppresses the apoptosis of human Sertoli cells. We have also identified that miR-100-3p binds to SGK3 in human Sertoli cells. Therefore, miR-100-3p controls human Sertoli cell proliferation and apoptosis by targeting SGK3. As such, this study offers new mechanisms by uncovering epigenetic regulators for determining the fate determinations of human Sertoli cells. Given the functional importance of miRNAs in mediating human reproduction, our study could provide novel targets for gene therapy of male infertility.

## Data Availability Statement

The original contributions presented in the study are included in the article/Supplementary Material, and further inquiries can be directed to the corresponding author/s.

## Author Contributions

BL performed the experiments, analyzed the data, and wrote the manuscript. YC, WC, LD, CL, and CW performed the experiments. ZH designed the study, analyzed the data, and wrote the manuscript. All authors contributed to the article and approved the submitted version.

## Conflict of Interest

The authors declare that the research was conducted in the absence of any commercial or financial relationships that could be construed as a potential conflict of interest.
