# Supplementary material for: Hsa-miR-100-3p Controls the Proliferation, DNA Synthesis, and Apoptosis of Human Sertoli Cells by Binding to SGK3
Source: Front Cell Dev Biol. 2021 May 11;9:642916. doi: 10.3389/fcell.2021.642916 (PMC8144512; doi:10.3389/fcell.2021.642916)
Supplement: Supplementary file 1 [file Table_1.DOCX]

**Supplementary Table 1. The Sequences of Gene Primers Used for RT-PCR**

| **Genes** | **Primer Sequences** |
| --- | --- |
| *WT1* | F：CGAGAGCGATAACCACACAACG  R：GTCTCAGATGCCGACCGTACAA |
| *GATA4* | F：GCGGTGCTTCCAGCAACTCCA  R：GACATCGCACTGACTGAGAACG |
| *GDNF* | F：CGCCGAAGACCGCTCCCTCG  R：ATCCATGACATCATCGAACTGATC |
| *SCF* | F：AATCCTCTCGTCAAAACTGAAGG  R：CCATCTCGCTTATCCAACAATGA |
| *FSHR* | F：TCTGCTGGTTCTGTTTCA  R：CATTCCTTGGATGGGTGT |
| *SOX9* | F：AGGAAGCTCGCGGACCAGTAC  R：GGTGGTCCTTCTTGTGCTGCAC |
| *AR* | F：CCTTCACCAATGTCAACTCC  R：CCACTGGAATAATGCTGAAGAG |
| *FGF2* | F：TCCTTTCTCCCTCGTTTCTTC  R：GATGTTTCCCTCCAATGTTTC |
| *GAPDH* | F：AATCCCATCACCATCTTCC  R：CATCACGCCACAGTTTCC |
| *SGK3* | F: CTGTAGATTGGTGGTGCCTTGGG  R: TGGACCAGGCTGTAAGACTCACTC |
