# Supplementary material for: Hsa-miR-100-3p Controls the Proliferation, DNA Synthesis, and Apoptosis of Human Sertoli Cells by Binding to SGK3
Source: Front Cell Dev Biol. 2021 May 11;9:642916. doi: 10.3389/fcell.2021.642916 (PMC8144512; doi:10.3389/fcell.2021.642916)
Supplement: Supplementary file 2 [file Table_2.DOCX]

**Supplementary Table 2. Information on Primary Antibodies Used for Immunocytochemistry and Western Blots.**

| **Antibodies** | **Vendors** | **Sources** |
| --- | --- | --- |
| WT1 | Abcam | rabbit |
| WT1 | Santa Cruz Biotechnology | rabbit |
| GDNF | Abcam | rabbit |
| SOX9 | Millipore | rabbit |
| SCF | Santa Cruz Biotechnology | rabbit |
| GATA4 | Santa Cruz Biotechnology | rabbit |
