# Supplementary material for: Hsa-miR-100-3p Controls the Proliferation, DNA Synthesis, and Apoptosis of Human Sertoli Cells by Binding to SGK3
Source: Front Cell Dev Biol. 2021 May 11;9:642916. doi: 10.3389/fcell.2021.642916 (PMC8144512; doi:10.3389/fcell.2021.642916)
Supplement: Supplementary file 3 [file Table_3.docx]

**Supplementary Table 3.** The sequences of miRNA mimics and inhibitors

| **Genes** | **Sequences** | |
| --- | --- | --- |
|  | **Sense (5'-3')** | **Antisense (5'-3')** |
| hsa-miR-100-3p mimics | CAAGCUUGUAUCUAUAGGUAUG | UACCUAUAGAUACAAGCUUGUU |
| hsa-miR-7974 mimics | AGGCUGUGAUGCUCUCCUGAGCCC | GCUCAGGAGAGCAUCACAGCCUUU |
| hsa-miR-29b-1-5p mimics | GCUGGUUUCAUAUGGUGGUUUAGA | UAAACCACCAUAUGAAACCAGCUU |
| hsa-miR-100-3p inhibitor | CAUACCUAUAGAUACAAGCUUG |  |
| hsa-miR-7974 inhibitor | GGGCUCAGGAGAGCAUCACAGCCU |  |
| hsa-miR-29b-1-5p inhibitor | UCUAAACCACCAUAUGAAACCAGC |  |
